# Surface Chemistry–Driven Oxidation Mechanisms in Ti_3_C_2_T_*x*_ MXenes

**DOI:** 10.1002/smsc.202500209

**Published:** 2025-06-24

**Authors:** Bradlee J. McIntosh, Bence G. Márkus, Anna Nyáry, Ferenc Simon, László Forró, Dávid Beke

**Affiliations:** ^1^ Stavropoulos Center for Complex Quantum Matter Department of Physics and Astronomy University of Notre Dame Notre Dame IN 46556 USA; ^2^ Department of Physics Institute of Physics and ELKH‐BME Condensed Matter Research Group Budapest University of Technology and Economics Műegyetem rkp. 3 Budapest H‐1111 Hungary; ^3^ Institute for Solid State Physics and Optics HUN‐REN Wigner Research Centre for Physics Budapest H‐1525 Hungary; ^4^ Kandó Kálmán Faculty of Electrical Engineering Óbuda University Tavaszmező u. 17 Budapest H‐1084 Hungary

**Keywords:** energy conversion, MXene, oxidation, Raman

## Abstract

Ti_3_C_2_T_*x*_ is a leading compound within the MXenes family and can find host in widespread applications. It is synthesized by selectively etching layers from the Ti_3_AlC_2_ precursor, and this process typically introduces surface terminations, T_*x*_, such as —OH, =O, or —F. However, the aggressive chemical conditions required for its preparation, as well as exposure to air, humidity, and heat, can lead to impurity phases that potentially compromise its desirable properties. Herein, a two‐step oxidation process is revealed during heat treatment, where initial oxidation occurs between layers without altering the integrity of the Ti_3_C_2_‐layered structure, followed by the formation of anatase TiO_2_ at elevated temperatures. The process is carefully monitored using *in situ* Raman spectroscopy and *in situ* microwave conductivity measurements, applied to Ti_3_C_2_T_*x*_ prepared using various etching techniques involving concentrated hydrofluoric acid, LiF + HCl, and HF + HCl mixtures. The oxidation process is heavily influenced by the synthesis route and surface chemistry of Ti_3_C_2_T_*x*_, with fluoride and oxyfluoride groups playing a pivotal role in stabilizing the anatase phase. The absence of these groups, in contrast, can lead to the formation of rutile TiO_2_.

## Graphical abstract

The oxidation of Ti_3_C_2_T_*x*_ MXenes follows a two‐step process, starting with diffusion‐controlled interlayer oxidation and exhibiting surface‐dependent characteristics. A fluoride‐rich surface facilitates the formation of anatase TiO_2_, while an oxygen‐rich surface favors the development of rutile.

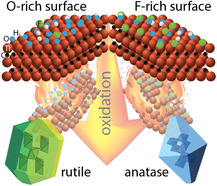

## 1 Introduction

Research into novel 2D materials, such as MXenes, is beneficial to enable and improve novel devices that are otherwise inaccessible using conventional materials, e.g., wearable, flexible electronics,^[^ ^1^, ^2^ ^]^ thanks to their unique atomic‐layered structures and tunable properties. These materials hold great promise for advancing technologies in energy storage, electronics, catalysis, and environmental remediation.

MXenes are formed by selectively etching the A layers from the M is a transition metal, A is an A group element (13‐14) X is C, N, B (MAX) phases,^[^ ^3^ ^]^ leading to a structure of M_*n*+1_X_*n*_T_*x*_, where M is an early transition metal (e.g., titanium, vanadium, chromium, molybdenum), A is an element, primarily from groups 13 and 14 (e.g., aluminum, silicon, or gallium), and X is carbon, nitrogen, or both. Furthermore, *n* can be 1, 2, or 3 and T_*x*_ represents surface terminations that are typically —OH, =O, or —F. These materials have emerged as leading candidates for a multitude of advanced technological applications due to their tunable electrical, mechanical, and surface properties.^[^ ^4^, ^5^, ^6^ ^]^ The potential to tailor these positions MXenes at the focus of research across diverse fields such as energy storage, catalysis, and electromagnetic interference shielding.^[^ ^6^, ^7^, ^8^ ^]^ Moreover, MXenes may also find applications in the field of nanoantenna‐based sensorics^[^ ^9^, ^10^ ^]^ because of their exceptionally good microwave and THz absorption capabilities. However, one of the most significant challenges limiting their widespread adoption is their inherent susceptibility to oxidation, which can severely compromise their structural integrity and reduce their performance even under ambient conditions and especially under elevated humidity.^[^ ^11^, ^12^, ^13^, ^14^ ^]^

The oxidation of MXenes, particularly for Ti_3_C_2_T_*x*_, typically results in the formation of titanium dioxide (TiO_2_), which detrimentally alters their properties by increasing electrical resistivity and reducing the number of functional surface sites.^[^ ^13^, ^15^ ^]^ Understanding the mechanisms driving this oxidative degradation is therefore critical for developing strategies to enhance MXene stability. Previous studies have demonstrated that oxidation is influenced by various environmental factors such as exposure to air,^[^ ^16^ ^]^ moisture,^[^ ^17^ ^]^ and light,^[^ ^13^ ^]^ with oxidation often initiating at defect sites or edges where TiO_2_ nanoparticles preferentially form. A recent theoretical study demonstrated that the nature of the surface terminations—such as —H, —OH, and —O significantly influences the interaction strength between Ti_2_CT_*x*_ surface and the formed TiO_2_.^[^ ^18^ ^]^

Nonetheless, the controlled oxidation of MXenes has been shown to confer beneficial properties in certain contexts. For instance, the formation of specific TiO_2_ polymorphs, such as anatase and rutile, can enhance the electrochemical performance of MXenes in supercapacitors and batteries.^[^ ^19^, ^20^ ^]^ In both applications, phase purity of the formed oxide is a crucial factor. Once a connected crystalline film of TiO_2_ is formed on the surface of the Ti_3_C_2_, one can differentiate two possible scenarios. 1) Anatase TiO_2_ has a crystal structure consisting of distorted edge‐sharing TiO_6_ octahedra. This structure features a 3D zig‐zag pathway in the crystal lattice, allowing Na^+^ and Li^+^ to diffuse along various directions.^[^ ^21^, ^22^ ^]^ 2) In contrast, rutile TiO_2_ forms 1D ion diffusion paths along its *c*‐axis. As an anode material, rutile is considered to have lower electrochemical activity; however, the Li^+^ diffusion coefficient along the *c*‐axis in rutile TiO_2_ is extremely high (in the order of 10^−6^ cm^2^ s^−1^)^[^ ^22^ ^]^ that gives direction selectivity for ion transport.

Moreover, the possible presence of titanium suboxides and Magnéli phases offers intriguing possibilities for applications requiring mixed valence states and high electrical conductivity on the surface,^[^ ^23^, ^24^ ^]^ which is also promoted by the already high conductivity of the underlying MXene structure. Given the critical importance of the TiO_2_ phase in these applications, a detailed understanding of the oxidation process, as well as the ability to control it, is essential for optimizing the properties of MXene‐based materials and compositions.

To gain further insights into the oxidation processes, we investigated Ti_3_C_2_T_*x*_ synthesized through various etching routes, focusing on the influence of heating by either using a laser beam or introducing direct heating. The evolution of the material was monitored by *in situ* Raman spectroscopy using laser‐induced heating and verified by following the stepwise oxidation in *in situ* microwave conductivity measurements applying a direct heat flow. Recently, the first approach was used to monitor the oxidation of Ti_3_C_2_T_*x*_ MXenes synthesized via the LiF‐HCl etching.^[^ ^25^ ^]^ We, however, focus on Ti_3_C_2_ prepared by different routes and monitored the beginning of the oxidation carefully to reveal its reaction kinetics. Our findings reveal a two‐step oxidation mechanism and TiO_2_ phase affinity that is highly dependent on the conditions of preparation and surface chemistry, with significant implications for the controlled synthesis of TiO_2_@C composites and the stabilization of specific TiO_2_ polymorphs.

## 2 Results and Discussion

### 2.1 Synthesis and Characterization

**Figure 1:**
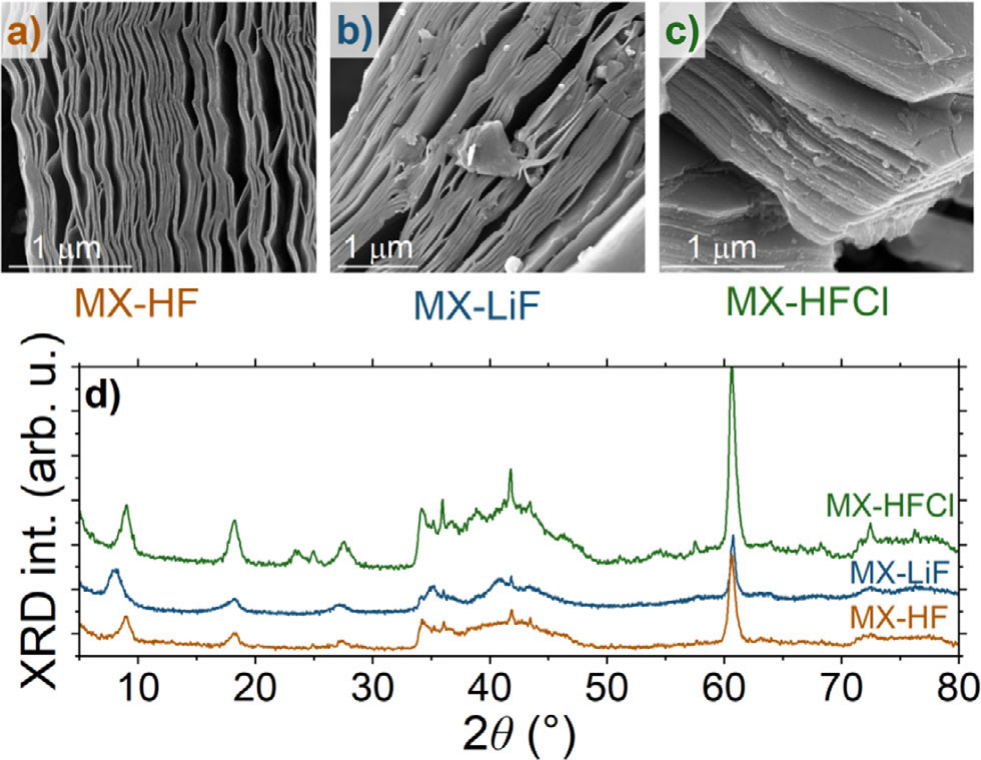
a–c) SEM images and d) XRD data of Ti_3_C_2_T_*x*_ synthesized through three different methods: concentrated HF (MX‐HF), LiF‐HCl solution (MX‐LiF), and HF‐HCl mixture (MX‐HFCl). The scale bars in the SEM images are all 1 μm.

For the synthesis of Ti_3_C_2_T_*x*_ MXenes, three different approaches, adapted from previous reports,^[^ ^26^, ^27^ ^]^ were used: etching in concentrated hydrofluoric acid (HF) (48%, “MX‐HF”), LiF‐HCl solution (“MX‐LiF”), and HF‐HCl mixture (“MX‐HFCl”). Delamination was also performed on a certain amount of MX‐LiF material using ultrasound sonication (“MX‐LiF del”). Characterizations of the resulting MXenes were carried out using X‐Ray diffraction (XRD), scanning electron microscopy (SEM), and elemental analysis, which confirmed successful etching and the formation of the characteristic accordion‐like layered structure (**Figure** **1**).

### 2.2 Effects of Annealing

Signs of oxidation is apparent when the samples were annealed. Here, we present measurements on samples annealed at 400 °C in air or in vacuum for 2 h to compare laser heating with conventional annealing. The samples annealed in air are labeled as “400‐Air”, and the vacuum‐annealed samples are labeled as “400‐Vac”. The microscopic examination, Raman, and X‐Ray photoelectron spectroscopy (XPS) measurement showed differences between the samples after annealing based on the type of synthesis method used.

**Figure 2:**
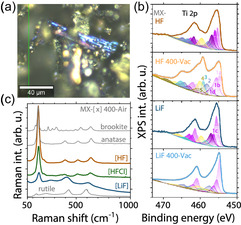
a) Microscope image of an MX‐LiF particle with layered oxidation after annealing in air. b) Ti 2p high‐resolution XPS spectra of different MXenes before and after vacuum annealing. Notations follow the suggestion of Natu *et al*.^[^ ^28^ ^]^ Figure 1a–d corresponds to the surface Ti atoms with +1 oxidation states and with three C—Ti bonds. The remaining possible bindings are O/O/O, O/O/F, O/F/F, and F/F/F, meaning that the Ti atom can bind to 1–3 oxygen or fluorine atoms. Peaks labeled with **2** and **3** are Ti^2+^ and Ti^3+^, respectively, peaks **4** and **5** are Ti^4+^. Peak **4** is dedicated to TiO_2_, while **5** is Ti^4+^, having Ti—F bonds. c) Raman spectra of air‐oxidized samples of all synthesis methods compared to the spectra of the rutile, anatase, and brookite phases of TiO_2_.

Two primary conclusions can be drawn from these measurements. First, the MX‐LiF sample demonstrates enhanced oxidation resistance compared to MX‐HF and MX‐HFCl. This is evidenced by the inhomogeneous oxidation patterns and layered structures observed in microscopic images (**Figure** **2**a), as well as by the lower intensity of TiO_2_‐related features in the XPS spectra of vacuum‐annealed specimens (Figure 2b). Indeed, deconvolution of the Ti 2p XPS peaks, based on the methodology proposed by Natu *et al*.^[^ ^28^ ^]^ reveals the presence of multiple oxidation states, including Ti^3+^ and Ti^4+^. Notably, the vacuum‐annealed samples also display distinct Ti—F bonds, and a significant reduction in highly fluorinated Ti—C bonds. Together, with increased peak intensities related to TiO_2_ and TiO_*x*_—F/TiF_4_ for MX‐HF 400‐Vac, this suggests that annealing plays a crucial role in modifying surface chemistry and, consequently, the oxidation behavior.

Second, the TiO_2_ phase that forms under annealing in air depends strongly on the etching route. HF etching predominantly yields anatase, whereas LiF‐based etching favors rutile TiO_2_. This conclusion is substantiated by the Raman spectra shown in Figure 2c. For clarity, they are shown together with the three most common phases of TiO_2_: rutile, anatase, and brookite. All samples, except MX‐LiF 400‐Air, contained exclusively the anatase phase of TiO_2_. MX‐LiF 400‐Air, in contrast, oxidized to a mixture of rutile and anatase TiO_2_ phases, with greater structural integrity and lower background photoluminescence compared to other samples.

### 2.3 Following the Oxide Growth *in Situ*

**Figure 3:**
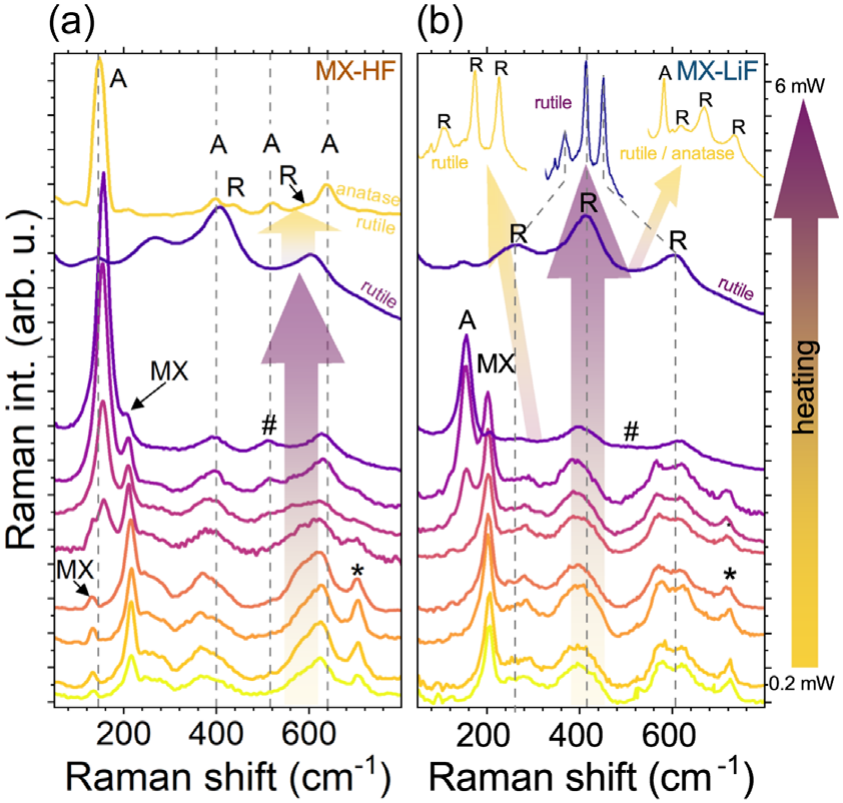
The laser‐induced oxidation of a) MX‐HF and b) MX‐LiF. MX‐HF oxidizes to an anatase phase which transforms into rutile at higher temperatures. The rutile phase transforms back to anatase when the sample cools down. MX‐LiF, in contrast, transforms into rutile when it cools down or is heated further. Arrows with transition color indicate the temperature changes between measurements, heating or cooling. # marks the position of the anatase $A_{1g}$ mode around 512 cm^−1^ and * marks the disappearing MXene peak around 720 cm^−1^. Peak positions of anatase and rutile TiO_2_ are labeled as A and R, respectively.

Given that *ex situ* characterization indicated that oxidation behavior is strongly influenced by the synthesis history, we conducted *in situ* Raman and microwave conductivity measurements to gain deeper insight into the underlying oxidation mechanisms and to clarify the specific pathways governing the formation of various oxide phases. **Figure** **3** presents the evolution of the Raman spectra of MX‐HF and MX‐LiF as the laser power was increased from 200 μW (18 kW cm^2^) to 6 mW (550 kW cm^−2^), effectively heating the samples. Here, the optical irradiance, given in parentheses, is calculated based on the Airy disc diameter of 1.09 μm^2^. Oxidation typically begins at ≈ 600 μW (55 kW cm^−2^) for MX‐HF and MX‐HFCl, and around 1 mW (92 kW cm^−2^) for MX‐LiF, MX‐LiF del, and MX‐HF 400‐Vac. Moreover, we can identify two distinguishable behaviors that exhibit similar features over all samples: 1) MX‐HF and MX‐HFCl, and 2) MX‐LiF, MX‐LiF del, MX‐HF 400‐Vac, and MX‐LiF 400‐Vac. Consequently, we focus our subsequent discussion on MX‐HF, MX‐HF 400‐Vac, and MX‐LiF.

In the case of the MX‐HF sample (group i), shown in Figure 3a, the first observable change is the emergence of a new peak at 157 cm^−1^, accompanied by a simultaneous decrease in the 720 cm^−1^ peak. As the laser power, and by extension the temperature as well, increased, the low‐wavenumber peak redshifted to 155 cm^−1^, and a new peak appeared at 512 cm^−1^. Although the changes in sample composition are evident in the Raman spectra from the emergence of the 512 cm^−1^ peak that closely resembles the spectral signature of anatase, unambiguously identifying a single oxide phase (or other underlying chemical or physical transformations) remains challenging. Previous studies labeled all peaks around 155 cm^−1^ as the *E*_g_ phonon mode of anatase TiO_2_ in the context of MXene oxidation. However, this position represents a significant blueshift for this Raman mode, given that the first *E*_g_ peak of crystalline anatase TiO_2_ is reported in the literature at 144–147 cm^−1^.^[^ ^29^, ^30^ ^]^ Various factors, such as tensile strain, doping, oxygen deficiency, and the presence of Ti^3+^, can induce such a shift toward higher wavenumbers; nevertheless, most studies report blueshifted *E*_g_ values at lower wavenumbers.^[^ ^30^, ^31^, ^32^, ^33^, ^34^, ^35^, ^36^ ^]^

The other Raman‐active modes of rutile TiO_2_ are the $B_{1g}$ and $E_{g}$ modes at 400 and 640 cm^−1^, of which both overlap with the Raman signals of Ti_3_C_2_T_*x*_, and the $A_{1g}$ mode at around 515 cm^−1^. Although observing the latter (together with the intense *E*_g_ mode) suggests the formation of rutile, similarities between the Raman spectra of suboxides, and the blueshifted *E*_g_ mode make it difficult to rule out the presence of a highly disturbed anatase phase, or the presence of suboxides, such as Ti_2_O_3_ or Ti_3_O_5_, or one of the Magnéli phases.^[^ ^37^, ^38^, ^39^ ^]^ at the beginning of the oxidation. As the temperature increased further, the oxide transforms into rutile. Once the laser‐induced heating stopped, the rutile phase transformed to an oxide with a *E*_g_ peak at 144 cm^−1^, which is typically attributed to anatase.

The MX‐LiF (see Figure 3b) and vacuum‐annealed samples behave quite differently. First, the low‐wavenumber peak appeared around 154 cm^−1^ instead of 157 cm^−1^, without the previously identified peak shift changes at the beginning of the oxidation. In stark contrast to the evolution of the MX‐HF sample, the peak at the position of the anatase $A_{1g}$ phonon mode (denoted with #) was not visible during heating. Second, the final phase of the completed oxidation, depending on whether the laser‐induced heating is stopped before or after the rutile phase formation, was rutile or an anatase/rutile mixed phase (as suggested by Figure 3b).

### 2.4 Oxidation Kinetics

The oxidation processes (thermal or photooxidation), induced by gradually increasing the laser power, clearly indicate that HF‐etched and LiF–HCl‐etched samples stabilize different TiO_2_ phases—anatase and rutile, respectively. We remain cautious about attributing the initial oxidation state to anatase, not only due to the unusual blueshift of the *E*_g_ band but also because the appearance of the 157 cm^−1^ peak attests a time‐ and power‐dependent sigmoidal growth under constant laser irradiation. Specifically, this peak grows over 5–15 min at a fixed power and then saturates; once it has stabilized, additional increases in laser power are required to enhance its intensity. Intermediate probe measurements at a lower power of 200 μW (18 kW cm^−2^), performed between power‐increment steps, revealed a reduction in the 157 cm^−1^ peak intensity. However, as the laser power was increased to higher levels, the magnitude of this reduction became less pronounced. The observed decrease—or, in some cases, disappearance—of the 157 cm^−1^ peak upon lowering the laser power suggests that this newly formed phase is unstable in the early stages of oxidation.

**Figure 4:**
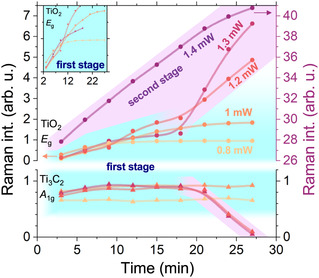
Peak intensities for MX‐HF samples as a function of exposure time at a constant laser power. The laser power was varied between 0.8–1.4 mW (73−128 kW cm^−2^). The first and second stages of the oxidation are highlighted with cyan and violet colors, respectively. The inset shows the enlarged region of the first stage with a similar slope and the sigmoidal growth characteristic.

A detailed examination of the initial oxidation period is presented in **Figure** **4** for MX‐HF. The response to sustained heating was monitored by following the TiO_2_ *E*_g_ and Ti_3_C_2_T_*x*_ $A_{1g}$ Raman peak intensities near 155 and 200 cm^−1^, respectively, under different fixed laser powers. The measurement revealed a two‐step oxidation mechanism. The TiO_2_ *E*_g_ peak initially rose, reaching a saturation level indicative of early‐stage oxide formation. At a critical power of around 1.3 mW (119 kW cm^−2^), the growth behavior transitioned to a more rapid, linear increase. The sigmoidal growth observed during the first stage is found to be temperature‐independent (see the inset in Figure 4), displaying the same slope at various temperatures and suggesting diffusion‐limited kinetics. At higher laser powers, the anatase‐like TiO_2_ peak growth evolved linearly over time, consistent with zero‐order reaction kinetics. The $A_{1g}$ MXene band (lower panel of Figure 4) remained unchanged during the diffusion‐limited oxide‐growth stage; it began to decrease only during the second stage of oxidation. This observation can be explained by the initial formation of a surface oxide that does not disrupt the Ti_3_C_2_ structure. In the second stage, oxide formation proceeds between the MXene layers, ultimately degrading the Ti_3_C_2_ backbone and reducing the corresponding Raman band intensity.

While the first oxidation stage seems to be temperature‐independent with a similar reaction constant, manifested in the temperature‐independent slope of the TiO_2_ *E*_g_ peak intensity growth, the temperature increase drives the reaction forward. This cannot be explained with a simple diffusion‐controlled reaction. It signals that activation is necessary for the diffusion or for other processes, leading to oxidation. However, the activation temperature cannot be extracted from the *in situ* Raman measurements. Therefore, the so‐called cavity perturbation technique^[^ ^40^, ^41^ ^]^ was utilized to obtain more information about the oxidation process by monitoring the microwave conductivity as a function of temperature. This technique measures the shift in frequency regarding the resonance frequency of the empty cavity, $f-f_{0}$, and the *Q*‐factor, $Q=f_{0}/\Delta f\propto L^{-1}$ of the microwave cavity with and without the presence of the sample. Here, Δf is the half‐width at half‐maximum of the cavity resonance response, which is related to microwave loss, *L*.

**Figure 5:**
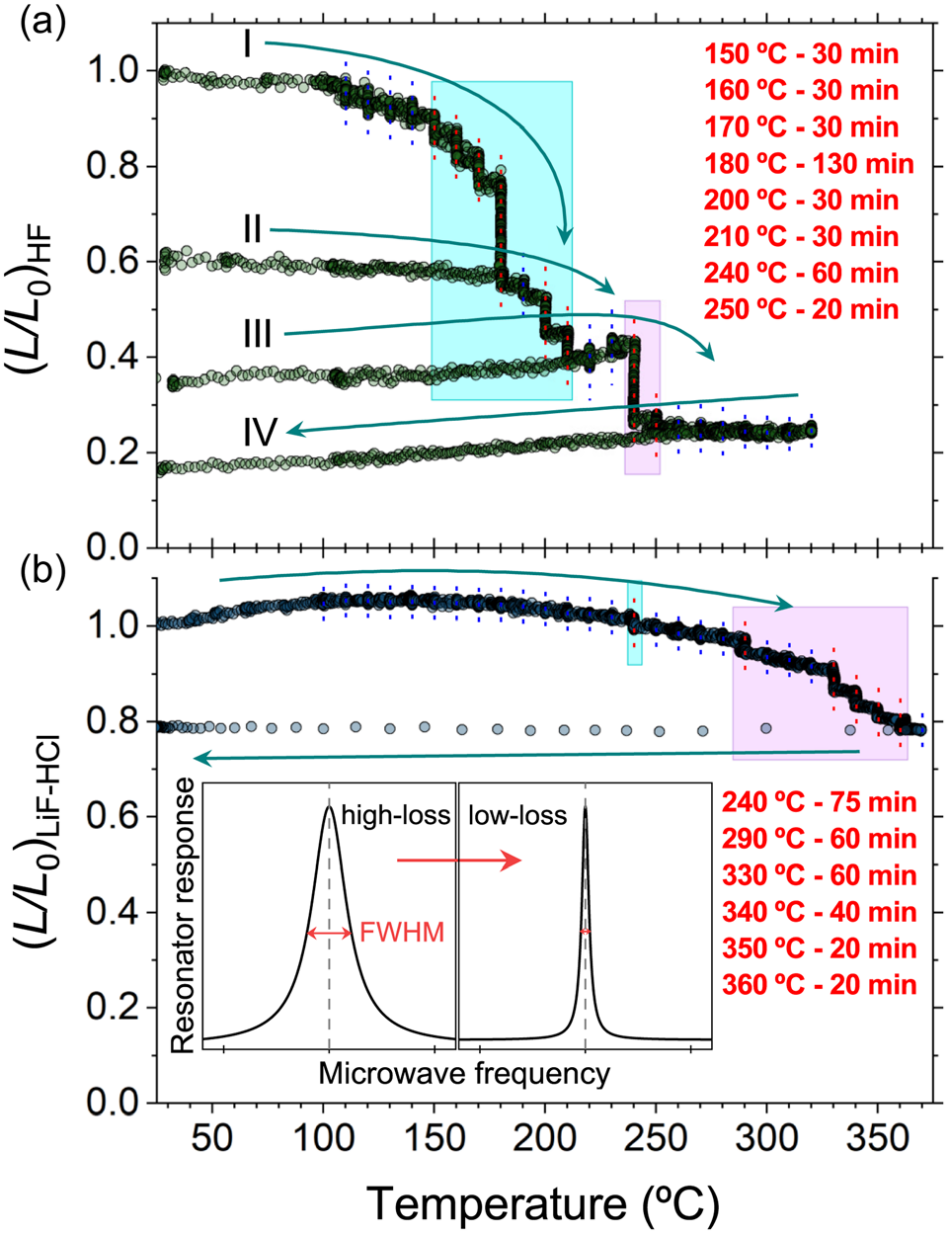
Normalized microwave loss, $L/L_{0}$, against the temperature of a) the MX‐HF and b) MX‐LiF samples measured by microwave conductivity measurements where $L_{0}$ is the microwave loss of the pristine material at room temperature. Both samples were heated while open to air. Colored vertical dashed lines show steps, where the temperature was held for a time given in the legend. Red (blue) lines denote where (no) significant change is observed. In the case of the HF‐etched sample, oxidation begins as low as 150 °C and is complete by 260 °C, whereas the LiF‐HCl‐etched sample is resistant up to 240 °C and for a fully oxidized state 360 °C is required. For the MX‐HF sample, the experiment was carried out in three heating segments, indicated by Roman numerals, after each the sample was cooled down to room temperature. The final cooldown is labeled as segment IV. Cyan (I)‐ and violet (II)‐shaded areas indicate the two stages of the oxidative transformation. Note the gradual change in the slope indicating that the material is transforming from a mostly metallic state to a doped semiconductor phase (assuming L∼σ justified by literature observations). The bottom inset illustrates the change in the observed microwave resonance induced by changes in the sample.

As presented in the inset in **Figure** **5**b, the response of the microwave cavity changes in line with a decreased microwave loss. This technique is only sensitive to the conductivity of individual particles as eddy currents are induced on a granular level. In other words, it is not influenced by the resistivity of the grain boundaries and is suitable for detecting small changes in the conductivity of the materials *in situ*.^[^ ^42^, ^43^ ^]^ The technique is proven to be a versatile tool to characterize powder materials without the need for physical contacts.^[^ ^44^ ^]^

The temperature‐dependent microwave conductivity measurements performed on MX‐HF and MX‐LiF are presented in Figure 5. The measurements also showed stepwise transformations in the materials that can be linked to the two‐stage oxidation observed in the Raman study. The temperature was continuously increased to 110 °C followed by 10 °C increments. At each temperature step, the temperature was held constant for at least 10 min and until no further change in the *Q* factor was observed before proceeding. In the case of the MX‐HF sample, two intermediate cooldowns were also performed to check the conductive behavior between the oxidative stages. The samples were open to air, allowing oxidation to occur. Both materials were stable up to 140 °C. Above this temperature, the MX‐HF material started to oxidize in multiple steps, which can be distinguished into two temperature ranges, similarly to the Raman results. The first stage of oxidation is between 150 and 210 °C followed by a second stage between 240–250 °C. Based on the initial for probe conductivity results (Figure S2, Supporting Information), we assumed that the measurements were in the Q∼ρ regime throughout the process, which is equivalent to L∼σ, where *σ* describes the microwave conductivity of the material. This is supported by previous literature results showing metallic temperature‐dependence.^[^ ^45^, ^46^, ^47^ ^]^ Consequently, the MX‐HF sample is transformed from metallic (segments I and II) to a doped semiconductor (segments III and IV) during the multiple step oxidation. No further transformation is observed up to 320 °C. The color of the material gradually changed from silvery black to yellowish white.

In stark contrast, the MX‐LiF is resistant up to 240 °C, where the first stage of oxidation can be observed. The next stage began at 290 °C and is completed around 360 °C. Interestingly, apart from the room temperature region (25–100 °C), this material did not exhibit a change in the conduction characteristic and remains mostly metallic. A visible change of color was also observed for the MX‐LiF sample, however, not as dramatic as in the case of the HF‐etched sample. Here, the final product was brownish. These are all probably the results of a difference in surface groups and oxidation pathway underlined by the Raman experiments.

In addition to the temperature‐dependent changes in the microwave loss, the cavity perturbation technique also yields the frequency shift with respect to the empty cavity resonance. The frequency shift data shows the same stepwise features, albeit less pronounced, for both materials (not shown) in agreement with the microwave loss results. In conclusion, the microwave conductivity measurements align well with the oxidation behavior observed with Raman spectroscopy. The heat treatments of Ti_3_C_2_T_*x*_ MXenes cause changes in the material, which occur in multiple steps. Similarly to the laser‐induced oxidation, the changes in microwave conductivity can be grouped into two separate regimes. Based on the Raman and microwave conductivity measurements, we propose that the two identified stages correspond to a heat‐induced oxidation.

We also note that both materials tend to absorb microwaves, as the at room temperature *Q*‐factor is remarkably lowered to about 1150 (MX‐LiF) and to 4500 (MX‐HF), compared to the unloaded cavity with $Q_{0}\approx 8000$ (with using the same amount of about 8 mg materials for each experiment). Comparing the two, we found that the MX‐LiF sample is a gradually better absorber which is in agreement with literature findings.^[^ ^48^ ^]^ This underlines the potential use of MXenes as excellent radio‐frequency and microwave absorbing materials for a range of applications.

### 2.5 Origin of the Oxide Phase Stabilization

The oxidation processes (thermal or photooxidation), induced by gradually increasing the laser power, clearly demonstrate that HF‐etched and LiF–HCl‐etched samples stabilize different TiO_2_ phases—anatase and rutile, respectively. Since the main differences among MXenes produced by different synthesis routes lie in their surface terminations, these surface moieties likely play a significant role in driving TiO_2_ formation. However, it is not an easy task to identify the presence or absence of particular moieties or groups of moieties that promote either anatase or rutile formation during the oxidation of Ti_3_C_2_T_*x*_. Both XPS and Raman spectroscopy indicate a complex (surface) chemistry for MXenes in general, making peak deconvolution and identification extremely challenging. In contrast, thanks to the similar behavior of MX‐HF 400‐Vac, which formed from MX‐HF and MX‐LiF, a careful comparison of MX‐HF, MX‐HF 400‐Vac, and MX‐LiF can provide the answer to this question. The only difference in the sample preparation between MX‐HF and MX‐HF 400‐Vac is the annealing *in vacuo* at 400 °C, which transforms its oxide affinity from anatase to rutile. MX‐LiF, therefore, can be used as a reference for the change. The surface changes after annealing that are similar to that of MX‐LiF can be connected to the stabilization of the different TiO_2_ phases. Consequently, we investigated the factors responsible for the emergence of these distinct oxide phases.

**Figure 6:**
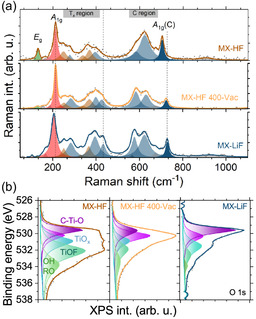
Deconvolution of the as‐prepared room‐temperature Raman spectra of the three different samples: a) MX‐HF, MX‐HF 400‐Vac, and MX‐LiF. The Ti_3_C_2_T_*x*_ $E_{g}$ and $A_{1g}$ modes, corresponding to the Ti—C vibrations, are highlighted in green and red, respectively. Peaks that remained unchanged after vacuum annealing are shown in brown, whereas those that transformed similarly to the MX‐HF spectrum are marked in blue. b) In the deconvoluted O 1s XPS spectra of MX‐HF, MX‐HF 400‐Vac, and MX‐LiF, the higher fluorine content in MX‐HF is clearly evident.

**Figure** **6**a presents the Raman spectra of the as‐prepared MX‐HF and MX‐LiF samples and the vacuum‐annealed MX‐HF 400‐Vac. The laser power was fixed to 200 μW (18 kW cm^−2^), and at this excitation power no heating occurs. The recorded broad Raman signals are deconvoluted by using Voigt functions. Numerous studies have attempted to interpret the Raman spectra of MXenes,^[^ ^25^, ^49^, ^50^, ^51^, ^52^, ^53^, ^54^, ^55^, ^56^, ^57^ ^]^ and we do not wish to challenge these interpretations here. Nevertheless, in the Raman spectrum of Ti_3_C_2_T_*x*_, the low‐frequency modes at around 130 cm^−1^ ($E_{g}$) and 200 cm^−1^ ($A_{1g}$) originate from in‐plane and out‐of‐plane vibrations of the outer Ti–C layers, whereas the high‐frequency modes at ≈ 600 cm^−1^ ($A_{1g}$) and 620 cm^−1^ ($E_{g}$) are associated with similar vibrational patterns within the central C layers. However, these theoretical zone‐center eigenmodes only partially match the experimentally observed Raman spectra. In practice, the aforementioned $E_{g}$ and $A_{1g}$ modes, as well as the peak above 700 cm^−1^, can be linked to zone‐center modes of MXenes, whereas the broader features spanning 220–700 cm^−1^ likely reflect a weighted average of phonons across the entire Brillouin zone. Both the relative abundance and local arrangement of surface species significantly affect the resulting Raman signatures, influencing not only the surface region but also the zone‐center modes.

We labeled in brown the peaks that remain largely unchanged after annealing (peaks in MX‐HF was not changed significantly due to the annealing), whereas those that resembled the post‐annealing peaks of MX‐LiF appear in blue. The Ti_3_C_2_‐related $E_{g}$ and $A_{1g}$ modes are highlighted in green and red, respectively.

The first notable difference that can be observed is a sharp peak at 131 cm^−1^ in the MX‐HF spectrum, which is nearly absent in the other two. The presence of this mode is commonly associated with resonance enhancement. However, because MXenes are metallic, the sample should theoretically always be in a resonant condition. Nevertheless, the decrease in the intensity of the peak linked to the $E_{g}$ mode is the most pronounced in MX‐HF, regardless of the excitation wavelength (437, 532, or 630 nm). We found that the $A_{1g}$ mode is at 215 cm^−1^ for both MX‐HF and MX‐HF 400‐Vac and is at 205 cm^−1^ for MX‐LiF. Previous studies have reported that the redshift of the $A_{1g}$ mode in LiF‐etched samples correlates with reduced fluorine content.^[^ ^52^ ^]^ However, as noted earlier, vacuum annealing also reduces the F concentration but does not significantly harden the $A_{1g}$ mode, consistent with earlier findings.^[^ ^56^ ^]^ Though, the surface reconstruction due to the used vacuum annealing does not affect the $A_{1g}$ mode.

In addition to the reduced intensity of the $E_{g}$ mode, the most pronounced alteration is observed in the $A_{1g}$(C) mode, located at 705, 725, and 728 cm^−1^ in MX‐HF, MX‐HF 400‐Vac, and MX‐LiF samples, respectively. Indeed, this peak is known to be highly sensitive to surface terminations, and the observed blueshift can be explained by fluorine depletion and oxygen enrichment at the surface. However, theoretical calculations indicate that substituting —F with —OH causes a redshift, while increasing the oxygen termination leads to a blueshift.^[^ ^49^, ^51^ ^]^ Despite these nuances, both the experimental and theoretical findings suggest that elevated O termination is responsible for the softening of the $A_{1g}$(C) mode. It should be noted that, in our deconvolution, all spectra exhibit a peak at 705 cm^−1^, though this feature is insignificant in the MX‐HF 400‐Vac and MX‐LiF samples.

The Raman region influenced by surface states also showed notable changes. In our measurements, the peak at ≈ 620 cm^−1^ decreases upon annealing, whereas the one at roughly 580 cm^−1^ is increased, making this region closely resemble that of MX‐LiF. This spectral window is generally connected to C vibrations: the former peak is often attributed to OH or F terminations, while the latter is linked to O terminations. In addition, two other OH‐related peaks at around 280 and 430 cm^−1^ are found to increase after annealing in a vacuum to the intensity similar to that was found in MX‐LiF.

Elemental analysis by both energy‐dispersive X‐ray spectroscopy (EDS, see Supporting Information) and XPS revealed reduced F concentrations after vacuum annealing of HF samples and in LiF‐etched samples relative to MX‐HF, in agreement with previous studies. Although the deconvoluted Ti 2p XPS spectra showed decreased F‐related peaks following vacuum annealing, the difference between MX‐HF and MX‐LiF is smaller than one might expect based solely on F concentration. In the O 1s region, however, the F‐related bonds in MX‐HF 400‐Vac are significantly diminished to levels comparable to those observed in MX‐LiF.

These findings suggest that MX‐HF 400‐Vac resembles MX‐LiF mainly because of its lowered F content, which in MX‐HF appears largely bound to oxygen. Hence, the high F concentration (likely in the form of oxyfluorides or Ti–O–F rather than just C–Ti–F) appears to be responsible for stabilizing the anatase phase. Although rutile is the most stable polymorph of TiO_2_, anatase can exhibit greater stability at the nanoscale due to the interplay between surface and bulk energies, leading to a crossover in thermodynamic stability as particle sizes decrease at around 30 nm.^[^ ^58^, ^59^ ^]^ In contrast, the anatase phase is susceptible to transformation into the rutile phase under certain conditions, which can limit its practical applications. The stabilization of the anatase phase can be achieved through the introduction of fluoride and oxyfluoride compounds.^[^ ^59^, ^60^, ^61^, ^62^ ^]^ This phenomenon can explain why anatase is often detected at the beginning of the oxidation process and why anatase is the stable oxide phase in MX‐HF. When oxide particles are initially small, anatase formation is favored. In an oxyfluoride‐rich systems, the presence of F^−^ ions can stabilize the anatase phase even when the particles become larger. Without the stabilization provided by F^−^ ions, the formed oxide stabilizes in the rutile phase.

## 3 Summary

In this study, we systematically investigated the oxidation behavior of Ti_3_C_2_T_*x*_ MXenes, synthesized via different routes, under laser‐induced heating. Our *in situ* Raman and microwave conductivity measurements revealed a complex oxidation pathway governed by the surface chemistry of the MXenes. HF‐etched samples exhibit a fluorine‐rich surface, leading to the preferential formation of anatase TiO_2_ at lower temperatures and smaller particle sizes, especially in the presence of fluoride and oxyfluoride species. With increasing temperature, a phase transition to rutile occurs; however, upon cooling, this transition reverses due to the stabilizing effect of F^−^ ions. In the absence of oxyfluoride species, which can be achieved by either annealing HF‐etched MXenes *in vacuo* or applying LiF‐HCl or other synthesis methods resulting in low F content, rutile remains the thermodynamically stable phase. Removing these fluorides notably increases the oxidation resistance and promotes the formation of rutile TiO_2_.

Our findings underscore the critical influence of surface chemistry and synthesis conditions on the oxidation route and final oxide phase, highlighting the pivotal role played by fluoride and oxyfluoride moieties. Additionally, *in situ* monitoring of the oxidation process revealed a two‐step mechanism, characterized by an initially unstable oxide phase with a diffusion‐controlled growth. The identification of a two‐step oxidation mechanism, with initial surface oxidation followed by bulk oxidation, and the possibility of phase control provides new insights into the engineered synthesis of TiO_2_@C composites, functionalized MXenes, and other TiO_2_‐based nanostructures from MXenes. We confirmed our findings using microwave conductivity measurements that showed the two‐stage oxidation and gave approximate temperature ranges attributed to these stages.

The differences in the oxidation mechanisms between HF‐ and LiF‐HCl‐etched MXenes underscore both the similarities and distinctions in their surface chemistry. The presence of oxyfluorides alters the oxidation behavior of the material and can impact various surface modification strategies. Our study showed that through vacuum annealing, a HF‐etched sample can be further modified to have a surface resembling that of the LiF–HCl‐etched Ti_3_C_2_T_*x*_, improving its stability against oxidation.

## 4 Experimental Section

MX‐HF: 2 g of Ti_3_AlC_2_ powder was gradually added to 30 mL of 48% HF under continuous stirring. The mixture was maintained at 35 °C and stirred for 24 h. MX‐HFCl: 2 g of Ti_3_AlC_2_ powder was gradually added to 30 mL of an etchant solution consisting of 12 M HCl, deionized water, and 48 % HF in a volume ratio of 6:3:1, respectively. The suspension was stirred continuously at 35 °C for 24 h. MX‐LIF: 2 g of Ti_3_AlC_2_ powder was gradually added to 30 mL of 9 M HCl solution containing 1.78 M LiF (prepared by dissolving 2.4 g LiF). The mixture was stirred continuously at 35 °C for 24 h.

XRD diffractograms were obtained using a Bruker D8 Discover high‐resolution spectrometer, equipped with a Cu K*α* line, in the θ−2θ geometry.

SEM images were captured in vacuum using an FEI Magellan 400 microscope operated at 10 kV electrons in the Notre Dame Integrated Imaging Facility (NDIIF) at the University of Notre Dame.

XPS measurements were performed at the Materials Characterization Facility (MCF) at the University of Notre Dame, using a PHI 5000 Versa Probe II equipped with monochromatic Al K*α* X‐rays (15 kV, 25 W).

Raman measurements were performed using a WITec alpha300R confocal Raman microscope using a Zeiss LD EC Epiplan–Neofluar 50×/0.55 long working distance objective and with a 1800 lines mm^−1^ grating.

Microwave conductivity measurements were carried out using the cavity perturbation technique^[^ ^40^, ^41^ ^]^ in the transmission geometry utilizing a standard TE_011_ cylindrical cavity. An HP 83752B microwave sweeper was used as a source of radiation with the microwave power set to 10 dBm (10 mW). The transmitted microwave power is detected using an HP 8472 A crystal detector and acquired using a Tektronix TDS‐320 oscilloscope. Every point is calculated from a 96–256 average of rapid scans over the resonance of the microwave resonator. The mass of the samples was around 8 mg.

## Conflict of Interest

The authors declare no conflict of interest.

## Supporting information

Supplementary Material

## Data Availability

The data that support the findings of this study are available from the corresponding author upon reasonable request.
