# Supplementary material for: Surface Chemistry–Driven Oxidation Mechanisms in Ti3C2T x MXenes
Source: Small Sci. 2025 Jun 24;5(8):2500209. doi: 10.1002/smsc.202500209 (PMC12362798; doi:10.1002/smsc.202500209)
Supplement: Supplementary file 1 — Supplementary Material [file SMSC-5-2500209-s001.pdf]

Supporting Information A: Elemental composition

This Supporting Information provides information about the elemental composition of the prepared materials, as summarized Table I. and depicted in Fig. S1. Two techniques are used to determine the composition: XPS, which is more surface sensitive, and EDS, which is more bulk sensitive.

TABLE I. Elemental composition (in at %) obtained from XPS and EDS measurements. XPS results are listed first for each sample, followed by EDS results (when available).

Sample	Al	C	F	O	Ti	Method
MX-HF	7.86	50.39	8.25	18.18	15.33	XPS
MX-HF 400-Vac	7.94	54.51	6.67	16.72	14.16	XPS
MX-HF 400-Vac	7.08	15.36	19.89	18.87	38.8	EDS
MX-HFCl	4.19	26.60	21.58	24.05	23.60	XPS
MX-HFCl 400-Vac	4.63	14.73	5.92	51.95	22.77	XPS
MX-LiF	1.09	34.47	8.36	19.83	26.43	XPS
MX-LiF	2.73	23.00	6.81	19.40	47.99	EDS
MX-LiF 400-Vac	1.03	40.54	6.40	22.37	30.38	XPS

Tables II. and III. summarize contributions from different species of titanium and oxygen using XPS after deconvolution.

TABLE II. Peak contribution in different samples (% concentration) for the Ti 2p XPS peaks.

Ti 2p peak	MX-HF	MX-HF 400-Vac	MX-LiF	MX-LiF 400-Vac
Ti I	13.0	12.0	11.0	13.0
Ti II	18.0	13.0	21.0	25.5
Ti III	9.7	1.2	9.4	13.7
Ti IV	3.0	5.0	12.7	2.0
Ti^{3+}	3.0	9.7	1.5	3.8
Ti^{2+}	8.0	7.5	4.0	3.7
TiO_2	8.5	5.0	7.0	3.7
TiO_xF	7.5	4.1	3.89	1.17

TABLE III. Peak contribution in different samples (% concentration) for the O 1s XPS peaks. Rows for **TiOx** and **TiOF** are highlighted in bold.

O 1s Peak	MX-HF	MX-HF 400-Vac	MX-LiF	MX-LiF 400-Vac
C-Ti-O I	16.17	21.73	39.68	21.34
C-Ti-O II	10.92	30.35	20.90	42.16
**TiOx**	**19.63**	**15.68**	**25.31**	**21.33**
**TiOF**	**36.27**	**19.65**	**10.62**	**9.97**
OH / R-O	17.01	12.59	3.49	4.60

Supporting Information B: Four-point resistance of MX-HF pressed pellet

Fig. S2 shows the resistance of the MX-HF sample in the temperature range of $22-110\,^{\circ}$C. A metallic behavior is observed, justifying the $Q \sim \rho$ assumption made in the main article for the microwave conductivity measurements. The material was compressed into a 3.2 mm diameter pellet and contacted with indium metal to ensure low contact resistance. In total, four contacts were placed on the surface of the pellet to employ a four-point resistance measurement. For wiring $50\,\mu$m 99.995% gold wires were used. The sample was measured then in a helium flow of 2.5 L/min to avoid oxidation. A Keithley 2400 SourceMeter was used as a current source and the voltage was measured by a Keithley 2182A Nanovoltmeter between the two innermost contacts.

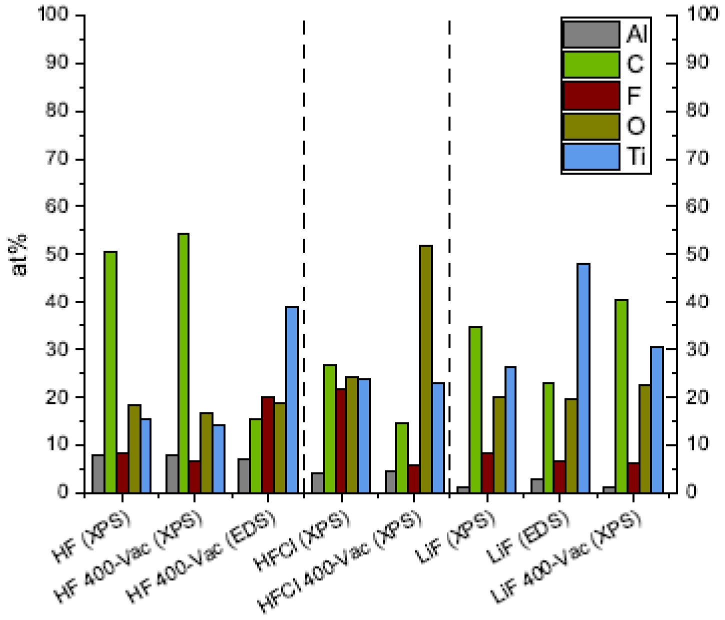

FIG. S1. Elemental composition of the investigated materials using XPS (surface sensitive) and EDS (bulk sensitive).

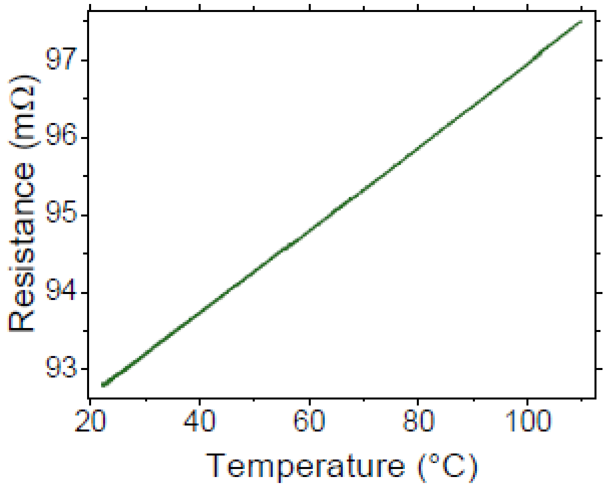

FIG. S2. 4-point resistance measurement of the MX-HF sample in the temperature range of $22-110$ $^\circ$C. The measurement was carried out in a continuous helium flow to avoid oxidation. The material clearly exhibit a metallic character and justifies the $Q \sim \rho$ assumption of the microwave conductivity measurements.
